# The impact of fresh gas flow on wash-in, wash-out time and gas consumption for sevoflurane and desflurane, comparing two anaesthesia machines, a test-lung study.

**DOI:** 10.12688/f1000research.13064.2

**Published:** 2017-12-22

**Authors:** Fredrik Leijonhufvud, Fredrik Jöneby, Jan G. Jakobsson

**Affiliations:** 1 Karolinska Institutet, Stockholm, Sweden; 2 Institution for Clinical Science, Karolinska Institutet, Danderyds University Hospital, Stockholm, Sweden

**Keywords:** wash-in, wash-out, low-flow anaesthesia, fresh gas flow, inhalational anaesthetics, sevoflurane, desflurane

## Abstract

Low-flow anaesthesia is considered beneficial for the patient and the environment, and it is cost reducing due to reduced anaesthetic gas consumption. An initial high-flow to saturate the circle system ( *wash-in*) is desirable from a clinical point of view. We measured the wash-in and wash-out times (time to saturate and to eliminate the anaesthetic agent, AA), for sevoflurane and desflurane, in a test-lung with fixed 3 MAC vaporizer setting at different fresh gas flow (FGF) and calculated the consumption of AA. We tried to find an optimal flow rate for speed and gas consumption, comparing two anaesthesia machines (AMs): Aisys and Flow-i. Time to reach 1 minimal alveolar concentration (MAC) (wash-in) decreased (p<0.05) at higher flow rates (1 – 2 – 4) but plateaued at 4-4.8 l/min. The consumption of AA was at its lowest around 4-4.8 l/min (optimal flow) for all but the Aisys /desflurane group. Wash-out times decreased as FGF increased, until reaching plateau at FGF of 4-6 l/min. Aisys had generally shorter wash-in times at flow rates < 4 l/min as well as lower consumption of AA. At higher flow rates there were little difference between the AMs. The “optimal FGF” for wash-out, elimination of gas from the test-lung and circle system, plateaued with no increase in speed beyond 6 l/min. A fresh gas flow of 4 l/min. seems “optimal” taking speed to reach a 1 MAC ET and gas consumption into account during wash-in with a fixed 3 MAC vaporizer setting, and increasing fresh gas flow beyond 6 l/min does not seem to confirm major benefit during wash-out.

## Introduction

Low flow anaesthesia is associated with several benefits, reducing the heat loss caused by cold gases and improving humidification in the airways ^1^. It is also environmentally friendly, reducing the release of anaesthetic agents into the atmosphere, and lastly, it also reduces costs. The initial period needed to achieve a steady state is dependent on fresh gas flow and the vaporizer setting ^2^. Many different FGF schemes have been described for the wash-in ^3, 4^. The wash-out of the inhaled anaesthetic from the lungs and circle is also of importance, to facilitate a rapid awakening and recommencement of protective reflexes.

The aim of the present study was to measure the time required to reach stable end tidal 1 MAC anaesthetic ( *wash-in*), to measure gas consumption during wash-in and the time needed to eliminate the anaesthetic agent ( *wash-out*) for sevoflurane and desflurane in the entire gas reservoir in the machine, circle system and in an lung model representing a 4 litre functional lung capacity and a test lung with a maximal capacity of 1 litre, and to compare the impact of different fresh gas flows and anaesthesia machines.

## Methods

This study used a test-lung setup. It was conducted in the operating room with proper scavenging equipment and central gas supply at Danderyds hospital, Sweden.

Two standard anaesthesia machines were used: Flow-i ^R^ (Maquet Critical Care AB, Solna, Sweden) and Aisys ^R^ (GE Healthcare, Madison, WI, USA), and two anaesthetic agents: sevoflurane and desflurane, making up a total of four test groups. All standard safety measures were followed, including checking each machine for performance and leakage before every session, in accordance with the Instruction for use (IFU), to ensure a safe working environment.

A standard disposable adult circle system was used (GE patient circuit, adult, disposable, 1.8 m and 1.5 litre volume), including a Y-piece at the end of the tube. A humidity-filter (Humid-vent, filter compact A) was fitted on the Y-piece. The circle system also included a standard CO _2_ absorber (soda lime canister) even though no carbon dioxide was used in the experiments, because some of the anaesthetic agent (AA) may also be absorbed and therefore mimic clinical conditions better.

A test lung was assembled using the following equipment: The Maquet test-lung 190 (Maquet Critical Care AB, Solna, Sweden), tidal volume max 1 litre and internal volume of 0, was mounted on one of the tubes of another Y-piece. Two 2-liter Intersurgical reservoir bags were connected to a T-tube to the other tube of the Y-piece.

Measurements of FiAA and EtAA of sevoflurane and desflurane were done with a mainstream sensor (instead of the AM side-stream sensors). IRMA AX +, (Masimo Sweden AB, Danderyd, Sweden) with an accuracy of 0.15 vol. % was used. This allowed us to get more exact measurements without any delay or dilution of gases. The mainstream sensor was connected to an external computer via a USB port and the Gasmaster ^®^ (Masimo Sweden AB, Danderyd, Sweden) program was used to record our measurements.

During the entire experiment a fixed respiratory rate and tidal volume was used, set at 12/min and 400 ml respectively. The positive end-expiratory pressure (PEEP) was set at 5 cm H _2_O and the inspiratory/expiratory rate was set two 1:2.

Two direct injection vaporizers were used for the Flow-i (one for each AA) and two variable bypass vaporizers for the Aisys (one for each AA). The AAs were released at fixed vaporizer settings of 3 × MAC-value (6 % and 18 % for sevoflurane and desflurane respectively).

### Wash-in

The timer/data log was started at the same time as when the vaporizer was turned on. The wash-in time to reach 1 MAC (i.e. increasing the EtAA concentration from 0 to 1 MAC), adjusted for a 40-year-old, 70 kg male (2% for sevoflurane and 6% for desflurane), was measured three consecutive times for each fresh gas flow (FGF). MAC values taken from the companies’ summary of product characteristics ^1^. The FGF tested included 1, 2, 4, 4.8, 6 and 8 l/min, with a composition of 80% oxygen and 20% air for each of the AAs and anaesthesia machines.

### Wash-out

Wash-out (reducing EtAA to 0 MAC) was performed after each wash-in session of the test-lung/circle. This was done by turning off the vaporizer and setting the FGF to one of 5 (2, 4, 6, 8 and 10 l/min) on each anaesthesia machine (AM) and AA.

The same protocol was used for sevoflurane and desflurane as well as for Flow-i and Aisys and the results for each wash-in session were presented as a mean of 3.

### Gas consumption

Gas consumption was calculated using the known wash-in time, FGF, vaporizer settings (VA concentration) and the vapour/liquid quote for each AA: vapour (ml)/liquid (ml) = 184 and vapour (ml)/liquid (ml) = 210 for sevoflurane and desflurane respectively. It means that 1ml of liquid AA equals 184ml and 210ml of vaporized sevoflurane and desflurane, respectively ^5^.

                                       This gives the equation: AA consumption = $\frac{\frac{time(s)\times FGFx1000}{60}\times VA\;conc\;(vol\%)}{\left(vapor/liquidquote)\times 100\;(vol\%\right)}$

### Statistical analysis

SPSS 24 (IBM, Armonk, NY, USA) was used for statistical analysis. All wash-in data was presented as mean of 3 measurements with standard deviation (SD) calculated. The mean time to reach 1 MAC for the different FGF was compared among the Flow-i and Aisys AM using analysis of variance (ANOVA). To determine which of the mean FGFs differed significantly from each other, we also performed Tukey's post hoc test. A p< 0.05 was considered statistically significant.

## Ethical statement

This is a test model study. The research does not involve human participants and/or animals, and thus no informed consent has been requested. The set-up is entirely experimental and no human or animals have been exposed to anaesthetics, and thus no ethical review board assessment has been considered necessary, according to Swedish research regulations.

## Results

**Figure 1.**
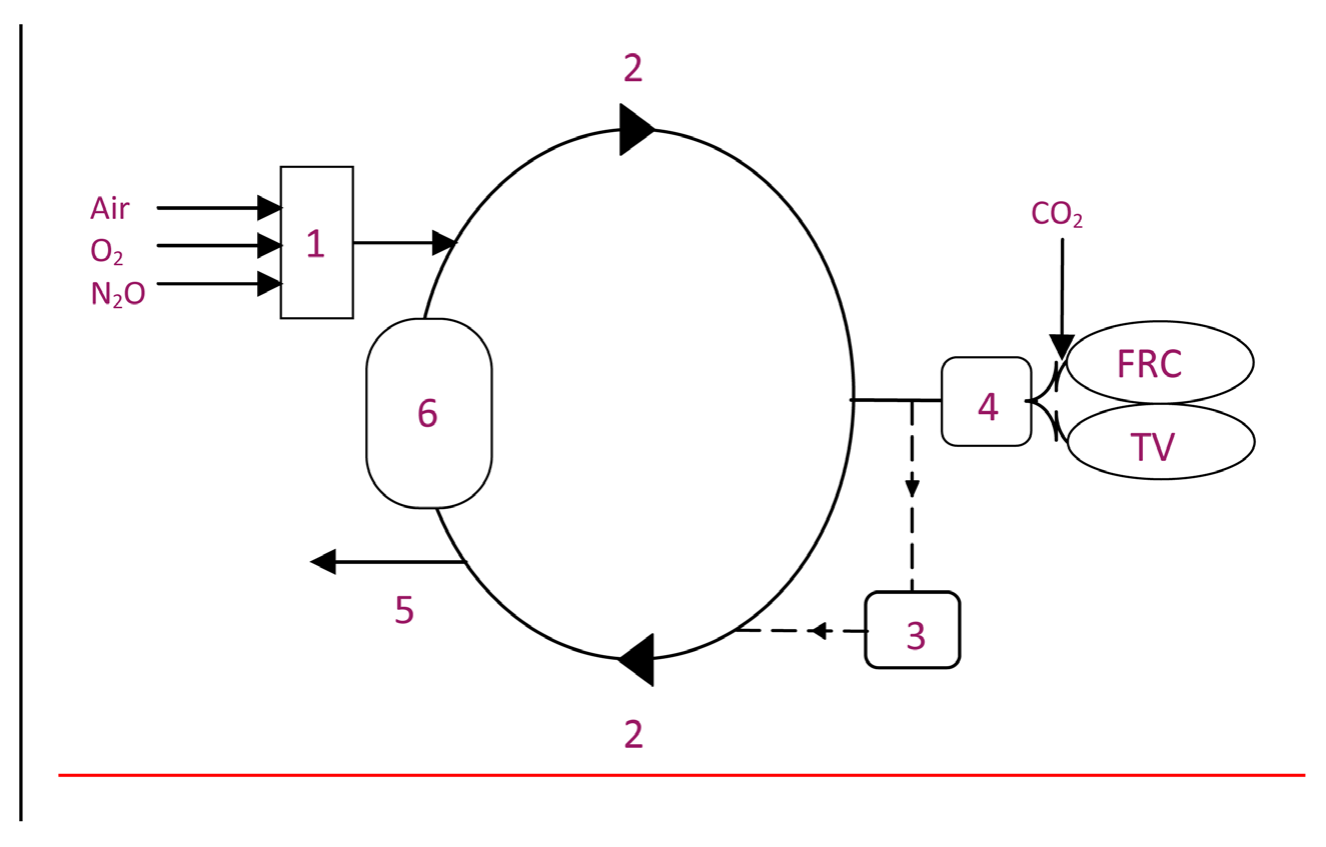
Schematic view of the experimental set-up. The fresh-gas flow is defined in this project as the gas-mixture introduced to the circle i.e. after the vaporizer. The functional residual capacity (FRC) consisted of two reservoir bags. The tidal volume (TV) consisted of one self-deflating bag. A small volume of gas gets diverted through the sidestream sensor but is reintroduced into the circle afterwards. 1. Vaporizer; 2. One-way valves; 3. Sidestream sensor; 4. Mainstream sensor; 5. Spill-valve; 6. CO _2_ Absorber.

**Figure 2.**
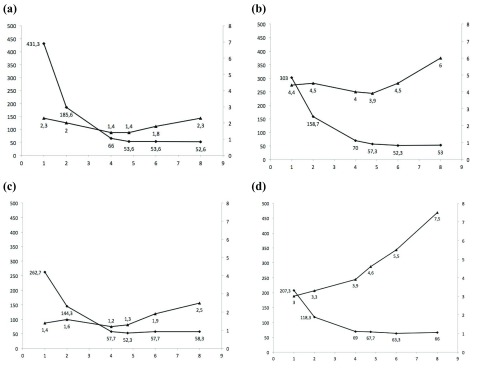
Time to reach 1 MAC and the calculated AA consumption for each fresh gas flows. ( **a**) Flow-i /sevoflurane. ( **b**) Flow-i /desflurane. ( **c**) Aisys /sevoflurane. ( **d**) Aisys/desflurane. Time(s) is on the Y-axis. FGF (l/min) is on the X-axis. AA (ml) is on the secondary Y-axis.

Wash-in times were significantly faster with higher flow rates for FGF spanning from 1, 2 and 4 l/min (p < 0.05) for all 4 groups ( Figure 1 and Figure 2). There was however a plateau starting at FGF 4–4.8 l/min, where increasing the FGF further up to 6 or 8 l/min did not shorten the time to reach 1 MAC significantly; similar patterns could be observed in all 4 groups. With Flow-i that plateau started at 4 and 4.8 l/min for sevoflurane and desflurane, respectively. With Aisys this plateau started at 4 l/min, for both the sevoflurane and desflurane group. Aisys had generally shorter wash-in times for both sevoflurane and desflurane at flow rates < 4 l/min. For flow rates of 4 l/min and above the difference was very small but somewhat more pronounced for desflurane wash-in, with Flow-i having shorter wash-in times than Aisys ( Figure 2 a–d).

Anaesthetic agent consumption showed a somewhat different pattern ( Figure 2 a–d ). The lowest consumption differed between sevoflurane and desflurane and also between the Aisys and Flow-i. The lowest consumption of AA per wash-in for Flow-i sevoflurane and Flow-i/desflurane, 1.4 ml and 3.9 ml respectively, was calculated at FGF equal to minute ventilation (4.8 l/min). The lowest consumption of AA for Aisys /sevoflurane, 1.3 ml, was calculated at a FGF at 4 l/min. The lowest consumption of AA for Aisys /desflurane, 3 ml, was calculated at the lowest FGF (1 l/min), in difference to the other 3 groups, followed by FGF 2 and 4 l/min with a calculated consumption of AA of 3.3 ml and 3.9 ml respectively. This was unique for Aisys /desflurane ( Figure 2 a–d).

**Figure 3.**
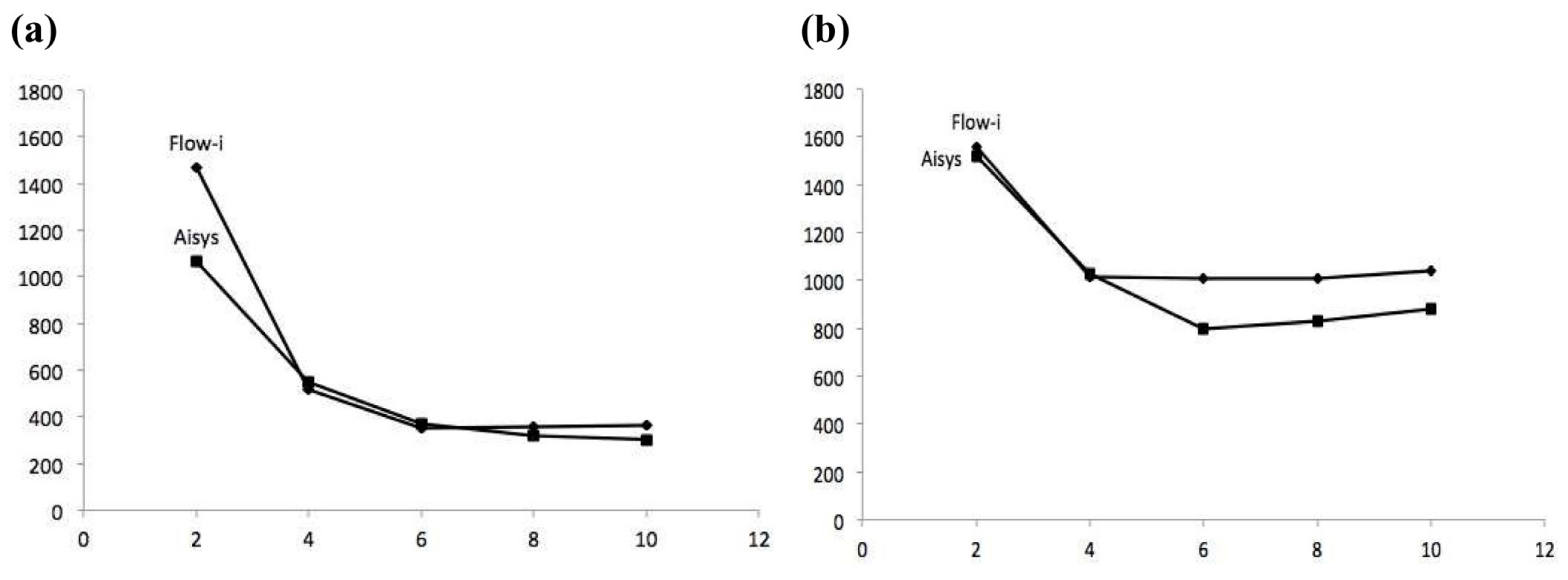
Wash-out times comparing Flow-i and Aisys AMs. ( **a**) sevoflurane ( **b**) desflurane. Time (s) is on the Y-axis and FGF (l/min) is on the X-axis.

Wash-out times were faster at higher FGF until a plateau was reached at around FGF 4–6 l/min. Increasing the FGF further did not lead to faster wash-out times, except for the Aisys / sevoflurane group. The fastest wash-out time with Flow-i /sevoflurane, 349s, was recorded at FGF 6 l/min where it also plateaued. Wash-out times for Flow-i /desflurane plateaued at FGF 4 l/min and 1013s, which differed a few seconds from the fastest wash-out time, 1007s, recorded at FGF 8 l/min. Wash-out times for Aisys /sevoflurane had a plateau that was not as distinct as the other groups with the fastest wash-out time, 303s, recorded at FGF 10 l/min. The fastest wash-out time for Aisys /desflurane, 797s, was recorded at FGF 6 l/min, where it reached a clear plateau ( Table 1). There was minor difference in elimination, Aisys being marginally faster ( Figure 3 a–b). No statistical analysis was performed on the wash-out data.

**Table 1.** Times at which wash-out plateaued, and fastest wash-out times at optimal flow rate.

	Flow-i	Aisys	All (mean)
**Sevoflurane**	349 s	317 s	333 s
**Desflurane**	1013 s	797 s	905 s
**All (mean)**	681 s	557 s	

Raw data for the measurements: Flow-I sevoflurane, Flow-I desflurane, Aisys sevoflurane and Aisys desflurane Click here for additional data file. Copyright: © 2017 Leijonhufvud F et al. 2017 Data associated with the article are available under the terms of the Creative Commons Zero "No rights reserved" data waiver (CC0 1.0 Public domain dedication).

## Discussion

The present study was set up to gain insight to whether two modern anaesthetic workstations with different vaporizer technologies and different internal volumes differed in time needed to saturate internal and externa gas reservoirs; circle system, CO2-absorber and further including an anticipated normal adult lung volume. We found, in this test-lung setup, that it is possible to increase the anaesthetic agent concentration from 0 to 1 MAC value in around one minute by using a FGF of 4–4.8 l/min on both AMs, but raising the FGF further did not result in shorter wash-in times. These flow rates were also the most efficient, in terms of both speed and gas consumption for both anaesthetic agents (sevoflurane and desflurane) and anaesthetic machines (Aisys and Flow-i) except when using Aisys and desflurane. Aisys had generally shorter wash-in times when using lower flow rates (< 4 l/min). For flow rates of 4 l/min and above, the difference was very small between the two machines tested. Anaesthetic agent consumption showed different patterns, but Aisys had generally lower gas consumption than Flow-i for both AAs. The shortest wash-out times were found at FGFs of 4–6 l/min; raising the FGF further did not result in shorter wash-out times. Wash-out of desflurane from the test-lung was more than twice as time-consuming as the sevoflurane wash-out.

This is merely a test-lung study and it was expected that there would be more rapid wash-in with increased fresh gas flow, but it is important to acknowledge the plateau at around 4–5 l/min FGF. Our results also show that Maplesons mathematical calculations of a theoretical optimal FGF at around 4 l/min were accurate ^6^. The result is also in line with what Shin *et al.* found in their study: increasing the FGF from low flow to moderately high flow will decrease the wash-in time ^7^. However, our results show that there seem to be no benefits to raising the FGF to levels of 6 l/min and above while performing a wash-in. This is an experimental test-lung set-up and we cannot comment to what extent the human uptake and elimination, e.g. the impact of the different blood gas solubility for the agents tested, would have on the time events studied.

Likewise the finding of increased AA consumption at both low and high FGFs is of interest, minimising the consumption of anaesthetic vapour is of economical as well as ecological importance. There was a difference between the AMs in terms of which FGF was most effective. We noted that the most effective FGF for the Flow-i, in terms of both consumption of AA and shortest wash-in time, was equal to the minute volume – 4.8 l/min. This applied to both sevoflurane and desflurane wash-in.

The small difference in wash-in between the AMs at lower fresh gas flows are in line with our previous study with a simpler test setup with sevoflurane ^8^. We used a more “physiologic” test-lung setup in the present study, which we believe mimics functional residual capacity and tidal volume for a 70 kg male. Our test-lung was constructed to represent both tidal volume (the volume of a normal breath) and the FRC in our test-lung setting, making the measurements more realistic. The Maquet test-lung 190 was representing the tidal volume and the two reservoir bags were representing a normal FRC (4 litre) in a 40-year-old male ^9^. We used an external main stream multi gas sensor in the present study, possibly reducing the difference in monitor performance. The difference in wash-in could of course be associated to the differences in vaporiser technology and algorithms for introduction of gas, a continuous influx or an intermittent – during inspiration only.

The finding that gas consumption is higher with desflurane than with sevoflurane may not be surprising, when taking the difference in MAC gas concentration into account. Whether there is any material absorption of desflurane in the Flow-i cannot be assessed. The huge difference, the twice as long time to eliminating desflurane as compared to sevoflurane, is to us also unexpected.

There are several limitations to this study, indeed this is merely a test-lung experiment and one may of course argue about its clinical application. The vaporizer setting was fixed at 3 MAC. We believe that our findings have clinical implications; we know that high fresh gas flows do not provide major benefits during wash-in or wash-out and there is the possibility to wash-in to a 1 MAC concentration within about a minute, 4 – 5 l/min FGF being the “optimal” time for wash-in. We used the AMC values presented on the product information sheet. There are slightly lower values suggested. Our study provides information around the machines, how the workstations performs during wash-in and wash-out of the gaseous phases. The uptake, blood solubility and likewise elimination must of course be taken into account. Both Aisys and Flow-i have built-in techniques for target end-tidal MAC, were fresh gas and vaporizers are automatically set to reach the target value ^10^. The Flow-i automatic gas control provides also the option to reach goal concentrations with different speed, possibly avoiding inappropriate heamodynamic response. We did study only high flow, rapid wash-in and wash-out. It would indeed be interesting to study also lower fresh-gas flows to assess whether there is differences in performance.

## Conclusions

Wash-in times, time to saturate internal and external gas reservoir including circle system, CO2 absorber and a test-lung volume of 4 litre, with fixed 3 MAC vaporizer settings decreased as the FGF increased, but plateaued at around flow rates of 4–4.8 l/min (optimal flow) on both AM. It is more effective, in terms of consumption of AA, to use optimal flow than lower or higher flow rates. Wash-out times plateaued at around flow rates of 4–6 l/min, higher FGF than that does not produce faster wash-out times. Further studies are required to confirm our findings in clinical practice, but also to study the end tidal target algorithms available on the AMs.

## Data availability

The data referenced by this article are under copyright with the following copyright statement: Copyright: © 2017 Leijonhufvud F et al.

Data associated with the article are available under the terms of the Creative Commons Zero "No rights reserved" data waiver (CC0 1.0 Public domain dedication). 

**Dataset 1: Raw data for the measurements:** Flow-I sevoflurane, Flow-I desflurane, Aisys sevoflurane and Aisys desflurane. DOI, 10.5256/f1000research.13064.d183806 ^11^

## Notes

^1^ www.baxterhealthcare.com.au/downloads/...pi/suprane_pi.pdf

www.baxterhealthcare.com.au/downloads/.../sevoflurane_pi.pdf
