# Supplementary material for: The impact of fresh gas flow on wash-in, wash-out time and gas consumption for sevoflurane and desflurane, comparing two anaesthesia machines, a test-lung study
Source: F1000Res. 2017 Dec 22;6:1997. Originally published 2017 Nov 13. [Version 2] doi: 10.12688/f1000research.13064.2 (PMC5754744; doi:10.12688/f1000research.13064.2)
Supplement: Peer review history

# Peer review: Referee response for version 2

**DOI:** 10.5256/f1000research.14708.r29258

**Published:** 2018-02-26

**Authors:** Moritz A. Kretzschmar, Thomas Schilling

**Affiliations:** 1 Department of Anaesthesiology and Intensive Care Medicine, Otto-von-Guericke-University Magdeburg, Magdeburg, Germany; 2 Hedenstierna Laboratory, Uppsala University, Uppsala, Sweden; 3 Department of Surgical Sciences, Hedenstierna Laboratory, Uppsala University, Uppsala, Sweden

Our questions and comments are addressed appropriately in the revised version. Attributing the differences in wash-in time to the different agent delivery technique is very plausible, and presents a further interesting field to study, although we do take notice that the differences are small and the clinical impact will probably be minute.

We have read this submission. We believe that we have an appropriate level of expertise to confirm that it is of an acceptable scientific standard.

# Peer review: Referee response for version 2

**DOI:** 10.5256/f1000research.14708.r29257

**Published:** 2018-01-10

**Authors:** Katarina Hallen

**Affiliations:** 1 Department of Anaesthesiology and Intensive Care Medicine, Institute of Clinical Sciences, The Sahlgrenska Academy, Gothenburg University, Gothenburg, Sweden

The most interesting is

 1. Sevo is much faster at FGF 2 in Aisys.

 2. Des is much faster at FGF> 4 in Aisys

 However, the results report is confused, as Table 1 indicates that it contains both times to fastest deployment and time to plateau, while the table only contains one time per machine and gas.

 Figure 1. Is it exactly the same principle of the circles in the two respirator machines? There should be 1 Figure for each machine, as the design of the circle may affect gas consumption.

 Fig.2. Put labels on all shoulders in the Figure instead of caption. Put headlines in the Figure to facilitate reading. In 2a, there should be Flow-I SEV, 2b Flow-I DES, in 2c Aisys SEV and 2d Aisys DES.

 Both Sevoflurane and Desflurane interfere chemically with the absorber, especially when dry, as in these experiments. This might give a certain difference in the consumption of gas. How does the authors comment on this? Is it exactly the same principle of the circles in the two respirator machines? There should be 1 Figure for each machine, as the design of the circle may affect gas consumption.

 Fig.2. Put labels on all shoulders in the Figure instead of caption. Put headlines in the Figure to facilitate reading. In 2a, there should be Flow-I SEV, 2b Flow-I DES, in 2c Aisys SEV and 2d Aisys DES.

 Both Sevoflurane and Desflurane interfere chemically with the absorber, especially when dry, as in these experiments. This might give a certain difference in the consumption of gas. How does the authors comment on this?

I have read this submission. I believe that I have an appropriate level of expertise to confirm that it is of an acceptable scientific standard.

# Peer review: Referee response for version 2

**DOI:** 10.5256/f1000research.14708.r29259

**Published:** 2018-01-04

**Authors:** Sirirat Tribuddharat, Thepakorn Sathitkarnmanee

**Affiliations:** 1 Department of Anesthesiology, Faculty of Medicine, Khon Kaen University, Khon Kaen, Thailand

The authors have addressed my concerns appropriately in the revised version. However, there are some issues that need additional revision. The authors should insert a reference for "1 MAC adjusted of a 40 year-old (2% for sevoflurane and 6% for desflurane)" in the Methods. The sentence "There are slightly lower values suggested." in the Limitations should be changed into "There are slightly different values suggested." since, according to the study of Nickalls RWD. BJA 2003; 91: 170-4, 1 MAC adjusted of a 40 year-old for sevoflurane and desflurane are both lower and higher, ie. 1.8% for sevoflurane and 6.6% for desflurane. Please also insert a reference for this sentence.

We have read this submission. We believe that we have an appropriate level of expertise to confirm that it is of an acceptable scientific standard.

# Peer review: Referee response for version 1

**DOI:** 10.5256/f1000research.14163.r28449

**Published:** 2017-12-15

**Authors:** Moritz A. Kretzschmar, Thomas Schilling

**Affiliations:** 1 Department of Anaesthesiology and Intensive Care Medicine, Otto-von-Guericke-University Magdeburg, Magdeburg, Germany; 2 Hedenstierna Laboratory, Uppsala University, Uppsala, Sweden; 3 Department of Surgical Sciences, Hedenstierna Laboratory, Uppsala University, Uppsala, Sweden

Thank you for the opportunity to review this interesting article. It is wonderful to see research on basic science addressing questions which are often taken for granted in clinical practice.

 We do have a few questions:

  

1. What is the clinical value of comparing sevoflurane and desflurane at the same MAC, if just the machines are of interest (and the solubility and clinical effects are negligible), would it not have been better to use the same vol%?
2. There are only two factors influencing wash-in to the circuit, and those are circuit volume and fresh gas flow. Setting aside the patient at the end of the anesthesia machine, this study evaluates the wash-in/-out of the total apparatus dead space. To better compare the measurements from the Aisys and the Flow-I, it would be necessary to reference the volumes (bag/volume reflector, internal tubing, respective soda lime canister etc.) of both machines up to the y-piece. We presume they were not identical, but by how much do they differ?
3. The wash-in time plateaued at around minute ventilation, which was to be expected, as both systems could be considered almost closed, with only minimal leakage. For the wash-out, the lowest FGF that eliminates rebreathing should indicate the plateau. Whereas with sevoflurane there is no apparent difference between the machines, reflecting the relationship between circle volume and FGF, the difference with desflurane is quite interesting. Could some materials used in the Flow-i have absorbed desflurane – We are thinking of the volume reflector or valves?
4. We cannot completely follow the data files: if the main stream sensor was placed after the y-piece, the high data points reflect Fi of the agent. Why was this position chosen and not the proximal (to the y-piece) end of the expiratory tube. This could have eliminated inspiratory/expiratory mixing in the acquisition, as the values were not recorded synchronized to the breath cycle.
5. An illustration of the experimental setup could enhance understanding for the reader.
6. The information of Figures 1 and 2 are redundant – they are repeated in Fig 3.

We have read this submission. We believe that we have an appropriate level of expertise to confirm that it is of an acceptable scientific standard, however we have significant reservations, as outlined above.

# Response

**Published:** 2017-12-16

**Authors:** Jan Jakobsson

**Affiliations:** Department of Physiology and Pharmacology, Karolinska Institutet, Sweden

**We do have a few questions:**


**  **

- What is the clinical value of comparing sevoflurane and desflurane at the same MAC, if just the machines are of interest (and the solubility and clinical effects are negligible), would it not have been better to use the same vol%?

*The aim of the study was to compare the anaesthetic works-station and the two third generation in haled anaesthetics, aiming for an similar end-tidal 1 MAC gas concentration and subsequently time to wash-out has from these gas volumes.*

 

- There are only two factors influencing wash-in to the circuit, and those are circuit volume and fresh gas flow. Setting aside the patient at the end of the anesthesia machine, this study evaluates the wash-in/-out of the total apparatus dead space. To better compare the measurements from the Aisys and the Flow-I, it would be necessary to reference the volumes (bag/volume reflector, internal tubing, respective soda lime canister etc.) of both machines up to the y-piece. We presume they were not identical, but by how much do they differ?

*The present study was set up to gain insight to whether the machine different in time need to saturate internal and externa has reservoirs, including an anticipated normal adult lung volume.*

 

- The wash-in time plateaued at around minute ventilation, which was to be expected, as both systems could be considered almost closed, with only minimal leakage. For the wash-out, the lowest FGF that eliminates rebreathing should indicate the plateau. Whereas with sevoflurane there is no apparent difference between the machines, reflecting the relationship between circle volume and FGF, the difference with desflurane is quite interesting. Could some materials used in the Flow-i have absorbed desflurane – We are thinking of the volume reflector or valves?

*We are not able to assess potential losses. Both machines are “tested” and approved for use with both sevoflurane and desflurane.*


* *

- We cannot completely follow the data files: if the main stream sensor was placed after the y-piece, the high data points reflect Fi of the agent. Why was this position chosen and not the proximal (to the y-piece) end of the expiratory tube. This could have eliminated inspiratory/expiratory mixing in the acquisition, as the values were not recorded synchronized to the breath cycle.

*The main-stream gas monitor was attached in parallel with in side-stream nipple. The readings, the recorded and presented values were the highest and stable values at each time-point.*

 

- An illustration of the experimental setup could enhance understanding for the reader.

*A graph of the set-up is added*


* *

- The information of Figures 1 and 2 are redundant – they are repeated in Fig 3.

*Deleted as suggested*

# Peer review: Referee response for version 1

**DOI:** 10.5256/f1000research.14163.r27918

**Published:** 2017-12-12

**Authors:** Sirirat Tribuddharat, Thepakorn Sathitkarnmanee

**Affiliations:** 1 Department of Anesthesiology, Faculty of Medicine, Khon Kaen University, Khon Kaen, Thailand

This report is interesting and may have clinical relevant in routine practice. However, the authors should address to the following points:

1. A figure showing the diagram of the circuit assembly including the test lung should be included. It’s quite confusing about the number of ‘Y-piece’. Normally, the test lung should be attached to the humidifier-filtered connected to a Y-piece. Why using 2 reservoir bags when normally only 1 is used in routine practice? Where is the location of the main stream sensor?
2. Please quote a reference for ‘1 MAC adjusted of a 40 year-old (2% for sevoflurane and 6% for desflurane). It’s quite different from “Nickalls RWD. BJA 2003; 91: 170-4”.
3. We have both anesthetic machines. The flowmeters on both Flow-i and AISYS are digitally controlled. They can be adjusted with accuracy of only 1 decimal place of litre, i.e., 0.1 L/min. Although the display on AISYS shows 2 decimal placed, i.e., 2.00 L/min, we can adjust flow of 0.1 L/min at a time only. How can the authors set FGF at 4.75 L/min?
4. The caption of Figures 3 and 4 does not match the figure, e.g., (a) in figure but (A) in caption.
5. The wash-in time derived from a test lung may not be inferred to human lung. The major confounding factor of wash-in time is anesthetic agent uptake from lung into human body ‘three compartments’ which affected by blood gas partition coefficient, cardiac output, organ tissue flow, organ tissue volume, and blood-tissue partition coefficient. That makes sevoflurane having longer wash-in time at same MAC and FGF. The test lung model did not take into account these factors. The authors should address to this point.
6. The wash-out time has the same problem. The test lung did not consider amount of anesthetic agent uptake into the body which has to be eliminated out of the body into account. Normally, at the same MAC, sevoflurane has longer wash-out time compared with desflurane because of higher blood-gas, blood-tissue and blood-fat solubility. The authors should address to this point.

We have read this submission. We believe that we have an appropriate level of expertise to confirm that it is of an acceptable scientific standard, however we have significant reservations, as outlined above.

## References

[1] NickallsRWMaplesonWW: Age-related iso-MAC charts for isoflurane, sevoflurane and desflurane in man. *Br J Anaesth*.2003;91(2) :170-4 1287861310.1093/bja/aeg132

[2] KapoorMCVakamudiM: Desflurane - revisited. *J Anaesthesiol Clin Pharmacol*.2012;28(1) : 10.4103/0970-9185.92455 92-100 10.4103/0970-9185.92455 22345954PMC3275981

[3] CowlesALBorgstedtHHGilliesAJ: Uptake and distribution of inhalation anesthetics agents in clinical practice. *Anesth Analg*.47(4) :404-14 5690531

# Response

**Published:** 2017-12-16

**Authors:** Jan Jakobsson

**Affiliations:** Department of Physiology and Pharmacology, Karolinska Institutet, Sweden

**This report is interesting and may have clinical relevant in routine practice. However, the authors should address to the following points: **

- A figure showing the diagram of the circuit assembly including the test lung should be included. It’s quite confusing about the number of ‘Y-piece’. Normally, the test lung should be attached to the humidifier-filtered connected to a Y-piece. Why using 2 reservoir bags when normally only 1 is used in routine practice? Where is the location of the main stream sensor?

*A graph of the set-up is added *

- Please quote a reference for ‘1 MAC adjusted of a 40 year-old (2% for sevoflurane and 6% for desflurane). It’s quite different from “Nickalls RWD. BJA 2003; 91: 170-4”.

*We used the FDA approved USP desflurane information sheet and Baxter Summary of Product characteristics; the 1 MAC for 40-45 years adult is set at 6 % (* www.baxter.com.pr/healthcare_professionals/.../suprane.html). *Likewise for sevoflurane we used the FDA and Baxter product documentations, stating 1 MAC at aye of 40 being 2.1 % but we used for this study the MAC 2 and 6 %. *

- We have both anesthetic machines. The flowmeters on both Flow-i and AISYS are digitally controlled. They can be adjusted with accuracy of only 1 decimal place of litre, i.e., 0.1 L/min. Although the display on AISYS shows 2 decimal placed, i.e., 2.00 L/min, we can adjust flow of 0.1 L/min at a time only. How can the authors set FGF at 4.75 L/min?

*The flow setting should be with 1 decimal, the 4.75 is incorrect for the Flow-I corrected*

- The caption of Figures 3 and 4 does not match the figure, e.g., (a) in figure but (A) in caption.

*Corrected excuse the edit mistake*

- The wash-in time derived from a test lung may not be inferred to human lung. The major confounding factor of wash-in time is anesthetic agent uptake from lung into human body ‘three compartments’ which affected by blood gas partition coefficient, cardiac output, organ tissue flow, organ tissue volume, and blood-tissue partition coefficient. That makes sevoflurane having longer wash-in time at same MAC and FGF. The test lung model did not take into account these factors. The authors should address to this point.

*This is indeed merely an experimental test-lung set-up and we cannot comment on the human uptake and elimination, e.g. the impact of the different blood gas solubility for the agents tested. *

- The wash-out time has the same problem. The test lung did not consider amount of anesthetic agent uptake into the body which has to be eliminated out of the body into account. Normally, at the same MAC, sevoflurane has longer wash-out time compared with desflurane because of higher blood-gas, blood-tissue and blood-fat solubility. The authors should address to this point.

*These, comments 6 & 7, are indeed one of limitations with our study, it is merely a test lung study. We have addressed the limitation in the discussion section, and we also clearly state that further studies are warranted confirming our results in the clinical setting. We still believe that our study provide information around the machines, how the workstations performs during wash-in and wash-out of the gaseous phases. The uptake, blood solubility and likewise elimination must of course be taken into account.*

# Peer review: Referee response for version 1

**DOI:** 10.5256/f1000research.14163.r27920

**Published:** 2017-11-20

**Authors:** Katarina Hallen

**Affiliations:** 1 Department of Anaesthesiology and Intensive Care Medicine, Institute of Clinical Sciences, The Sahlgrenska Academy, Gothenburg University, Gothenburg, Sweden

Thank you for asking me for a peer review.

 I have some questions to the authors regarding the study: The impact of fresh gas flow on wash-in, wash-out time and gas consumption for sevoflurane and desflurane, comparing two anaesthesia machines, a test-lung study. by J. Jakobsson.

  

1. Is there a difference between the two vaporizer used in the experiment?
2. Comment on the difference in a direct-injection and a bypass affect how respirators works.
3. Why the maximum inflow of the two gases? This is not clinically relevant. Would not it be better for another group with half the inflow, 4% and 8%?
4. Why non-parametric tests? Is this normal distribution of data?
5. The figures are not sufficient quality, high solution pictures please.
6. Explain in an educational way how you calculate gas consumption.
7. Do you have any theory why Aisys is faster at low flow with desflurane? Do you have any theories?
8. Why does it take longer to wash-out desflurane than sevoflurane in a mechanical lung model? This does not correspond to the clinical picture.
9. Does desflurane have lower density than sevoflurane? Is that why it takes longer to wash-out the desfluran? Can you come up with some theories about the most remarkable result in the study?
10. I can not find sevoflurane wash-out in Figure 4. 

I have read this submission. I believe that I have an appropriate level of expertise to confirm that it is of an acceptable scientific standard, however I have significant reservations, as outlined above.

# Response

**Published:** 2017-11-26

**Authors:** Jan Jakobsson

**Affiliations:** Department of Physiology and Pharmacology, Karolinska Institutet, Sweden

Thank you for valid comments. Please find below responses to your queries.

- Is there a difference between the two vaporizer used in the experiment?

Yes indeed both the technique to create the gaseous phase, the internal reservoir and the respiratory phase when gas is introduced into fresh gas. The Flow-i “injects gas” during inspiration while the Aysis has a more traditional vaporizer technique. The internal gas volume, reservoir is smaller in the Flow-i. This is the reason for our interest in comparing the machine, to assess how these different solution create difference during a rapid wash-in, gas bolus.

- Comment on the difference in a direct-injection and a bypass affect how respirators works.

The aim of the study was indeed to study how these different techniques act in a test-lung set up at different flow rates. It would also be interesting to study lower vaporizer settings at that study may be worth carrying out.

- Why the maximum inflow of the two gases? This is not clinically relevant. Would not it be better for another group with half the inflow, 4% and 8%?

The aim of this study was to study the bolus, high concentration performance. It would indeed be of interest to study lower setting.

- Why non-parametric tests? Is this normal distribution of data?

Traditional paremetric test was used, ANOVA test.

- The figures are not sufficient quality, high solution pictures please.

We will improve image

- Explain in an educational way how you calculate gas consumption.

The formula is derived from the reference.

- Do you have any theory why Aisys is faster at low flow with desflurane? Do you have any theories?

This may be an effect of the different vaporizer techniques and the phase during inspiration/expiration gas is delivered to the fresh gas.

- Why does it take longer to wash-out desflurane than sevoflurane in a mechanical lung model? This does not correspond to the clinical picture.

The finding is also to us somewhat surprising. One must acknowledge that this is merely the lung volume and circle wash-out. The low blodd gas solubility impacts the biological wash-out from the human body. It does not describe the elimination from volumes per see.

- Does desflurane have lower density than sevoflurane? Is that why it takes longer to wash-out the desfluran? Can you come up with some theories about the most remarkable result in the study?

The longer wash-out may be related merely to the huger amount of gas, the 6 % gas volume.

- I can not find sevoflurane wash-out in Figure 4. 

The Figure 4 A. presents sevoflurane wash-out.
